# Adipose-Derived Mesenchymal Stem Cell Administration Does Not Improve Corneal Graft Survival Outcome

**DOI:** 10.1371/journal.pone.0117945

**Published:** 2015-03-02

**Authors:** Sherezade Fuentes-Julián, Francisco Arnalich-Montiel, Laia Jaumandreu, Marina Leal, Alfonso Casado, Ignacio García-Tuñon, Enrique Hernández-Jiménez, Eduardo López-Collazo, Maria P. De Miguel

**Affiliations:** 1 Cell Engineering Laboratory, IdiPAZ, La Paz Hospital Research Institute, Madrid, Spain; 2 Ophthalmology Department, Ramon y Cajal Hospital Research Institute, Madrid, Spain; 3 Tumor Immunology Department and Innate Immunity Group, IdiPAZ, Madrid, Spain; University of Udine, ITALY

## Abstract

The effect of local and systemic injections of mesenchymal stem cells derived from adipose tissue (AD-MSC) into rabbit models of corneal allograft rejection with either normal-risk or high-risk vascularized corneal beds was investigated. The models we present in this study are more similar to human corneal transplants than previously reported murine models. Our aim was to prevent transplant rejection and increase the length of graft survival. In the normal-risk transplant model, in contrast to our expectations, the injection of AD-MSC into the graft junction during surgery resulted in the induction of increased signs of inflammation such as corneal edema with increased thickness, and a higher level of infiltration of leukocytes. This process led to a lower survival of the graft compared with the sham-treated corneal transplants. In the high-risk transplant model, in which immune ocular privilege was undermined by the induction of neovascularization prior to graft surgery, we found the use of systemic rabbit AD-MSCs prior to surgery, during surgery, and at various time points after surgery resulted in a shorter survival of the graft compared with the non-treated corneal grafts. Based on our results, local or systemic treatment with AD-MSCs to prevent corneal rejection in rabbit corneal models at normal or high risk of rejection does not increase survival but rather can increase inflammation and neovascularization and break the innate ocular immune privilege. This result can be partially explained by the immunomarkers, lack of immunosuppressive ability and immunophenotypical secretion molecules characterization of AD-MSC used in this study. Parameters including the risk of rejection, the inflammatory/vascularization environment, the cell source, the time of injection, the immunosuppression, the number of cells, and the mode of delivery must be established before translating the possible benefits of the use of MSCs in corneal transplants to clinical practice.

## INTRODUCTION

Corneal transplantation has been performed successfully for over 100 years, and it is the most common form of solid tissue transplantation in humans [1]. In the USA alone, approximately 26,000 corneal transplants are performed every year [2].

Unlike other solid organ transplantation, human leukocyte antigen (HLA) typing and systemic immunosuppressive drugs are not used, yet 90% of those considered normal-risk transplants such as first-time grafts in avascular graft beds and non-inflamed graft beds can survive 5 years after surgery [3]. However, this number decreases with time, to 43% corneal graft survival at 15 years for low-risk corneal dystrophies and 77% for keratoconus. These numbers become progressively important with the increasing age of the population worldwide.

Moreover, preoperative conditions known to abrogate immune privilege and that characterize high-risk grafts, such as vascularization of the graft-recipient bed, rejection of a previous graft, inflammation at the time of transplant, or atopy, increase the problem of survival of the corneal graft transplant. In these high-risk recipients, graft survival is even poorer: for herpetic eye, 72% survival is achieved at 5 years, and 49% at 15 years; for corneal ulcers, 48% survival at 5 years is reported and decreases to 21% at 15 years [4].

The acceptance of corneal allografts compared with other categories of allografts is known as immune privilege. Immune privilege is actively sustained by the expression of soluble and cell membrane molecules that can block the induction of immune response, deviate immune responses down a tolerogenic pathway, or inhibit the expression of effector T cells and complement activation [5]. However, some conditions dismantle the immune privilege of the corneal allograft and promote rejection, which remains the leading cause of corneal allograft failure [1]. Nevertheless, a high proportion of the human corneal allografts that undergo rejection are not perceived to be a high rejection risk pre-transplant. In these graft recipients, a post-transplant event leads to subversion of the immune privilege. These events include local episodes of alloantigen-independent inflammation, such as a loosened transplant suture, bacterial suture-associated infection, or herpetic infection recurrence.

Although topical corticosteroids remain the only immunosuppressive agents routinely used in corneal allograft recipients, in high-risk patients, systemic immunosuppressants such as calcineurin inhibitors, including cyclosporine and tacrolimus, or mycophenolate mofetil can prolong graft survival time [6,7]. However, therapeutic dosages are limited by drug toxicity and the potentially life threatening complications associated with immune suppression.

Other interventions are being attempted with the aim of restoring or augmenting immune privilege, and the use of mesenchymal stem cells (MSCs) is a promising approach [8]. In addition to their regenerative properties, MSCs have an immunoregulatory capacity and they elicit immunosuppressive effects both *in vitro* and *in vivo*. Not only are they immunoprivileged cells due to the low expression of MHC-II and costimulatory molecules on their cell surface, but they also interfere with the various pathways of the immune response by means of direct cell-to-cell interactions and soluble factor secretion [8]. MSCs inhibit the cell proliferation of T cells, B-cells, natural killer cells (NK) and dendritic cells (DC), producing what is known as division arrest anergy. Moreover, MSCs can stop a variety of immune cell functions: cytokine secretion and cytotoxicity of T cells and NK cells; B cell maturation and antibody secretion; DC maturation and activation; and antigen presentation.[8] Some *in vivo* studies on small rodent models have shown prolongation of corneal graft survival with the use of postoperative intravenous injection of MSCs isolated from bone marrow [9], but the *in vivo* tolerogenic properties of MSCs have been questioned in other rat organ transplant models [10,11].

In the present study, we investigated the effect of local and systemic injection of MSCs derived from adipose tissue (AD-MSC) into rabbit models of corneal allograft rejection with either normal- or high-risk vascularized corneal beds with the aim of preventing transplant rejection and increasing the duration of graft survival.

## MATERIALS AND METHODS

### Animals and experimental groups

For the normal-risk corneal transplant experiments, 36 inbred male New Zealand White rabbits, weighing 3 kg and older than 3 months, were used as recipients. A total of 18 inbred male Black Fox rabbits, weighing 2.5 kg and older than 3 months, were used as donors, in order to increase the failure rate of corneal graft survival by using two different rabbit strains. Four groups of 9 rabbits each were established. In the sham group, the rabbits underwent corneal transplantation, and immediately afterwards they were administered corneal stromal injections of vehicle. In the MSC-treated groups, the rabbits underwent corneal transplantation, and immediately afterwards they were administered a corneal stromal injection of human AD-MSCs (activated or not, see below) or rabbit AD-MSCs.

For the high-risk corneal transplant experiments, 16 inbred male New Zealand White rabbits, weighing 3 kg and older than 3 months, were used as recipients; and another 8 inbred male New Zealand White rabbits, weighing 3 kg and older than 3 months, were used as donors. Two groups of 8 rabbits each were established. Every rabbit underwent a prevascularization procedure (see below). In the AD-MSC group, the rabbits underwent corneal transplantation and 4 intravenous injections of AD-MSC were administered at days: d-7 (7 days before surgery), d0 (immediately after transplantation), d3, and d14-d15 (immediately after removal of the sutures). In the sham group, the rabbits underwent corneal transplantation and 4 intravenous injections of HBSS were administered at the same time points.

Only the right eyes of the recipient rabbits were subjected to corneal transplantation, thus no animal was blinded. All procedures involving rabbits were approved by the La Paz Hospital Animal Welfare Committee.

### Isolation of MSCs and PBMCs

Lipoaspirate from three female donor patients (ages 41 to 47, mean 44 years old) undergoing elective liposuction (BMI range 28,1 to 28,4, mean 28,3) was obtained by a plastic surgeon. Patients were healthy otherwise and received no drug therapy, except for one patient receiving fluoxetine for depression. The isolation protocols were approved by the Institutional Review Board of La Paz Hospital (Madrid, Spain) and were in accordance with the Declaration of Helsinki (2000) of the World Medical Association. Informed consent was obtained from patients undergoing elective liposuction. Active infection by HIV, hepatitis C virus, and syphilis was ruled out by serological analyses. AD-MSCs were isolated as previously described [12,13] with slight differences, and were stored in the biobank of La Paz Hospital. Briefly, the adipose tissue from human liposuction was washed with phosphate buffered saline (PBS) and digested with 0.09% collagenase I in PBS (Gibco-BRL, Grand Island, NY, USA) for 45 min at 37°C under gentle agitation. It was then inhibited with fetal bovine serum (FBS; Gibco) and centrifuged at 300g for 10 min to obtain the adipose-tissue stroma vascular fraction (SVF) of the pelleted cells and discard the floating adipocytes. The pellets were treated with erythrocyte lysis buffer (160 mM NH_4_Cl; 10 mM KHCO_3_; 1 mM EDTA; all from Sigma-Aldrich, St. Louis, MO, USA) for 15 min at room temperature (RT). The pellets were washed with PBS and seeded at 1x10^6^ cells per plate into 10-cm plates (Corning) containing standard media that consisted of Dulbecco’s Modified Eagle Medium (DMEM; Gibco), supplemented with 10% FBS, Na-pyr 110mg/L (Gibco), Glutamax 862 mg/L (Gibco), and 1% penicillin-streptomycin (Sigma). This protocol has been demonstrated to be effective in isolating h-AD-MSCs capable of multipotent lineage differentiation by a previous study of our group [14].

Likewise, lipectomy of retroperitoneal adipose tissue was performed on 4 inbred New Zealand White rabbits, which were used as adipose tissue donors. Isolation of the mesenchymal stem cells from the rabbit lipectomy followed a similar process with few differences: the rabbit adipose tissue was cut into pieces before being digested with 0.2% collagenase I. All *in vivo* experiments were performed using MSCs at passage 3–4.

Peripheral blood mononuclear cells (PBMCs) were obtained from buffy coats from donating blood samples supplemented with anticoagulants. Briefly, samples were diluted 1:1 with PBS and 35 ml carefully run over 15 ml Ficoll in a 50 ml conical tube. Samples were centrifuged at 400g for 40 minutes at 20°C without brake. The mononuclear cell layer was transferred to a new 50 ml tube and washed with PBS at 300g for 10 minutes at 20°C. The cell pellet was resuspended in 50 ml of PBS and centrifuged at 200g for 15 minutes at 20°C in order to remove platelets. Cells were cultured in RPMI+antibiotics without serum for 1h to remove adherent monocytes.

### Induction of chondrogenic, osteogenic and adipogenic differentiation

**Chondrogenic Induction**. A 10 μl drop of culture medium containing 8x10^6^ AD-MSC cells per ml of suspension was plated in normal medium [15]. Five hours later, the culture medium was replaced by a chondrogenic differentiation culture medium: DMEM, 1x insulin-transferrin-selenium (Sigma-Aldrich), 0.1 μM dexamethasone (Merck, Darmstadt, Germany), and 50 μg/ml 2-phosphate ascorbic acid (Fluka, Ronkonkoma, NY). Medium changes were performed 3 days a week for 4 weeks. Chondrogenic differentiation was confirmed using Alcian Blue staining at acidic pH to show production of sulfate proteoglycans.

**Osteogenic Induction**. AD-MSC cells were plated at 2 x 10^4^ cells per cm^2^ and cultured in normal medium for 24 h. Afterwards, the medium was changed to an osteogenic induction medium (adapted from [16]): DMEM, 10% FBS, 0.1 μM dexamethasone (Merck), and 50 μg/ml 2-phosphate ascorbic acid (Fluka). The medium was changed 3 days a week for 2 weeks. Osteogenic differentiation was confirmed by alkaline phosphatase activity detection using 1 mg/ml Fast Red-TR (Sigma-Aldrich) and 0.04% Naphthol AS-MX (Sigma-Aldrich).

**Adipogenic induction**. AD-MSC cells were seeded at a density of 3x10^3^ cells/cm^2^ in DMEM with 10% FBS. 24 h later, the medium was changed to adipogenic differentiation medium [17]: DMEM, 10% FBS, 500 μM isobutylmethylxanthine (IBMX, Sigma), 1 μM dexamethasone (Sigma) and 1 μM indomethacin (Sigma). A total of 10 μM of insulin (Actrapid, Novo Nordisk A/S, Bagsværd, DK) was added for 24 h every 3 days. Differentiation was maintained for 15 days, and then the presence of lipidic intracellular vacuoles was revealed by Oil Red O staining and Cole’s hematoxylin counterstaining.

### Flow Cytometry

To characterize hAD-MSCs, they were cultured to passage 3 and then resuspended in PBS and incubated for 30′ at 4°C with the following antibodies: phycoerythrin-conjugated anti-CD34, phycoerythrin-conjugated anti-CD90 (Abcam), fluorescein-conjugated anti-CD45, fluorescein-conjugated anti-CD105 (Chemicon), fluorescein-conjugated anti-IDO (R&D Systems), fluorescein-conjugated anti CD80 (Biolegend), phycoeritrin-conjugated anti CD40 (Abcam), Peridinin Chlorophyll Protein Complex (PerCP)-conjugated anti HLA-DR (Miltenyi) and Allophycocyanin-conjugated CD86 (Miltenyi). For intracellular markers (IDO) a previous permeabilization step was performed. For nitric oxide (NO) detection, a DAF-FM diacetate kit (Molecular Probes) was used. Briefly, detached cells were incubated with DAF-FM diacetate for 30′ (hMSCs) or 60′ (rbMSCs) at 37°C, followed by 30′ of PBS. Cells were then resuspended in PBS and analyzed in a flow cytometer (BD Biosciences, Franklin Lakes, NJ, USA) using the Cell Quest Pro program.

For AD-MSCs stimulation, they were cultured with 20 ng/ml IFN-γ and TNF-α for 4 h. Then the medium was changed and cells were cultured without cytokines for another 16 h prior to FACS analysis.

### MSCs-T lymphocytes coculture

We established MSCs-PBMCs cocultures at different MSCs-T cell ratios for 7 days in RPMI+5% FBS+antibiotics and with different stimulation regimes. Six experimental groups were set: negative control (PBMCs alone without T cell stimulation), positive control (PBMCs alone with T cells stimulation), unstimulated MSCs+unstimulated PBMCs, stimulated MSCs+unstimulated PBMCs, unstimulated MSCs+stimulated T cells and stimulated MSCs+stimulated T cells. All cocultures were established at MSC:T cell ratios of 1:1, 1:10 and 1:100 and in triplicate cultures for each ratio. For T cell stimulation, dynabeads T-activator CD3/CD28 kit (Gibco) was used following manufacturer’s instructions. MSCs were stimulated with 20 ng/ml of TNF-α and IFN-γ for 4h. Treatment with mitomycin C (10μg/ml) was used to cease MSCs proliferation. PBMSc were labeled with carboxyfluorescein succinimidyl ester (CFSE) and anti-CD3 flow cytometry (Immunostep) was performed in order to determine T cell percentage in the PBMCs pool. Proliferation rates were analyzed using flow cytometry for CFSE and CD3. A negative control of non-proliferating cells was used to set the peak of CSFE intensity. Percentages of T cell proliferation were established as the sum of CFSE peaks different in intensity from that of negative control.

### Cytokine detection

For cytokine detection, the BD Cytometric Bead Array (CBA) human inflammatory cytokines kit was used (BD Biosciences). AD-MSCs were cultured normally or activated for 4 h with INF-γ and TNF-α. Then, the medium was changed and cells were cultured for 16 h without cytokine supplementation. The supernatant was collected and the assay was performed following the manufacturer’s instructions. Briefly, a mix of the capture beads was added to each sample together with the human inflammatory cytokine PE detection reagent, and incubated for 3h at RT and protected from light. Samples were washed and then resuspended in wash buffer and analyzed in a flow cytometer (BD) using the Cell Quest Pro program. A standard curve was established following the manufacturer’s instructions to determine the concentration of each cytokine.

### Surgical procedure: Corneal transplantation

**Prevascularization**. The rabbits were anesthetized by intramuscular injection of medetomidine/ketamine (0.15 mg/kg and 10 mg/kg, respectively) and tramadol/midazolam (5 mg/kg and 1 mg/kg, respectively). The prevascularization was performed (modified from [18]), placing 2 concentric and continuous sutures in the cornea 14 days before transplantation, using an 8/0 blue virgin silk suture.

**Surgical procedure**. The rabbits were anesthetized by intramuscular injection of medetomidine/ketamine (0.15 mg/kg and 10 mg/kg, respectively) and tramadol/midazolam (5 mg/kg and 1 mg/kg, respectively) in the induction phase and were maintained with inhaled isoflurane. The center of the donor cornea was excised with a 7 mm trephine and the recipient graft bed was prepared with a 6.5 mm punch. The donor cornea was attached with 16 interrupted 10/0 nylon sutures. Two million MSCs in 300 μl of Hanks’ balanced salt solution (HBSS, Gibco) or vehicle alone in the control group were introduced within the corneal stroma with a 30G syringe in allogeneic groups. Likewise, the syngeneic groups were injected intravenously with 2 million MSCs resuspended in 1 ml of HBSS or vehicle alone in the control group at the aforementioned dates. The sutures were removed 14 days after transplantation. Opioids were administered subcutaneously and gentamicin eye drops were topically administered for 5 days after surgery. All the surgeries were performed by the same surgeon.

### Treatment with MSCs

The MSCs were harvested and labeled with a 1:200 dilution of the dialkylcarbocyanine fluorescent solution chloromethyl-benzamide (Vybrant CM-DiI, Molecular Probes) in D-PBS for 13 min at 37°C and washed twice in PBS to fluorescently label all intracellular membranes (the organelles) except the plasma and nuclear membranes.

For the stromal injection in the normal risk experiments, 2x10^6^ cells were resuspended in 300 μl of HBSS and were injected in the host cornea. In the control group, the same volume of HBSS was used. For the activated MSCs, TNF-α (Gibco) and IFN-γ (Gibco) (both at 20 ng/ml) were added to culture dishes 4 h before harvesting and applied in the same fashion. These molecules have been shown to stimulate MSC release of immunosuppressive molecules such as IDO and NO [19–21].

For the intravenous injection in the high-risk experiments, 2x10^6^ cells were resuspended in 1 ml of HBSS and injected into the ear veins at corresponding time points (-7d, 0d, 3d, and 14d). The injection of cells or vehicle was performed in a blind fashion on all experimental animals.

### Clinical evaluation

All the grafts were evaluated 3 times a week with slit lamp microscopy by an independent observer and in a blind fashion following an already established method [18]. Briefly, the time of rejection was calculated according to a rejection index (RI) representing the overall graft status. A scale of 0 to 12 was used, based on 3 criteria (haze, edema, and neovascularization), each of which was scored on a scale of 0 to 4. A graft was considered rejected once the RI reached 6 or when the haze was over 3 [18]. Indefinite graft survival was defined as survival of a clear graft for 8 weeks.

### Histopathology

The rabbits were euthanized at rejection time, or at 8 weeks if the graft showed indefinite survival. The cornea, lungs, spleen, and skin from the injection point were excised and either fixed in 10% formaldehyde and included in paraffin or frozen directly in isopenthane and embedded in OCT for cryosectioning.

The paraffin-embedded corneal tissue sections were observed under epifluorescence to identify the injected cells labeled with CM-DiI and were stained with hematoxylin and eosin where histopathological studies were performed. H&E stained samples were used to measure corneal thickness with J Image image analysis software.

### Immunohistochemistry

Rabbit corneas were fixed for 24 h in 3.7% formaldehyde and then paraffin-embedded. Three-μm thick sections were incubated with primary antibody anti rabbit-CD45 (Antigenix America) for 1h and then incubated with biotin-conjugated secondary antibody (1:100, Vector Labs, Burlingame, CA, USA) for 1h. Samples were then incubated with avidin-peroxidase complex (Vector), and reveled with DAB (Invitrogen) enhanced or not with Cl_2_Co. Leukocyte infiltration was measured by counting CD45+ cells in an area of 0,66 mm^2^ under a 40x objective and compared between groups.

### Leukogram

Blood from the rabbits that underwent intravenous injection was extracted once a week for the length of the experimental period and the levels of total leukocytes, lymphocytes, neutrophils and monocytes were determined with the Sysmex XT 2000 (Roche). The blood differential was established by observation under an optical microscope. A normal range for blood parameters in our rabbit population was previously established using the data from day-14 of the experiment, when the rabbits were untouched. A normal range was established as the mean ± 2 typical deviations.

### Statistical analysis

The Kaplan-Meier method and the Chi-squared test were used to compare the survival curve of the experimental groups. For the rejection index parameters, the Mann-Whitney method was used to compare two variables, and the Kruskal-Wallis method for multiple variables comparisons. Student’s t-test was used to determine differences among the corneal thickness, leukocyte infiltration numbers, and leukograms. All methods were assessed at a 0.05 level of significance.

## RESULTS

### AD-MSC characterization

**Fig 1:**
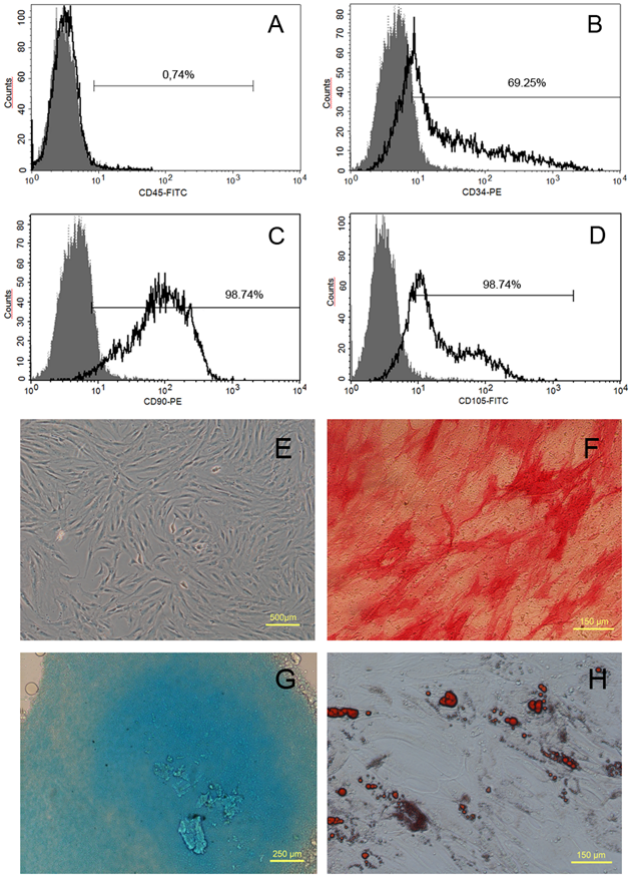
AD-MSC characterization. A-D: Fluorescence-activated cell sorting of a sample of the stromal vascular fraction of human adipose tissue for markers CD45, CD34, CD90 and CD105 (open lines) with respect to autofluorescence (shadow lines). Percentages of each population are given within the graph for each marker. E: Phase contrast of cultured living AD-MSC cells. Observe the normal fusiform morphology. F: In vitro osteogenesis of the human AD-MSC, revealed by alkaline phosphatase staining with Fast Red. G: In vitro chondrogenesis of the human AD-MSC cultured using the micromass technique and revealed by Alcian blue. H: In vitro adipogenesis of the human AD-MSC, revealed by Oil red staining in red.

AD-MSC showed mesenchymal stem cell features, such as absence of CD45, and presence of mesenchymal stem cells markers CD34, CD90 and CD105 as expected (Fig. 1A-1D). Purity based on CD90/CD105 expression was determined at more than 98% (Fig. 1D). Additionally, AD-MSC showed adherence to plastic, and trilineage (osteo-, condro- and adipo-) differentiation (Fig. 1E-1H).

### Normal risk grafts

**Fig 2:**
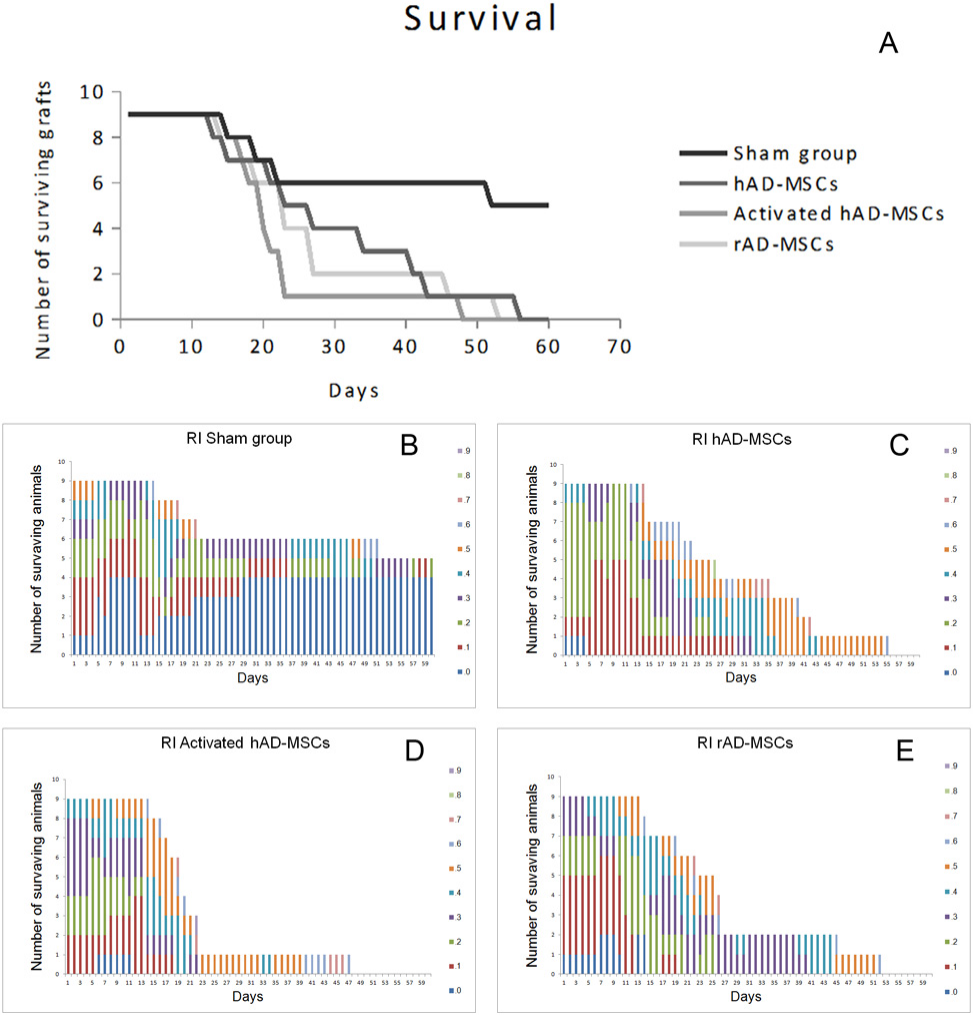
A: Survival curve of normal-risk corneal grafts. Graft survival was significantly shortened by stromal injection of any of the cell types, compared with vehicle injection, and graft survival was similar among the different cell-treated groups (p = .008): the sham group (rabbits treated with vehicle), the hAD-MSCs (rabbits treated with stromal injection of human adipose-derived MSCs), the activated hAD-MSCs (rabbits treated with stromal injection of previously IFN-γ and TNF-α activated human adipose derived-MSCs) and the rAD-MSCs (rabbits treated with stromal injection of rabbit adipose derived-MSCs). B-E: Rejection index score (haze, haze, edema, and neovascularization) comparison between groups. Different colors show different rejection index scores in the surviving grafts at any time point.

In the sham group (rabbits subjected to transplant surgery but receiving only vehicle), 5 of 9 rabbits survived beyond the follow-up period, with a two-month survival rate of 55% (median 60%) (Fig. 2A).

In the rabbits that underwent transplant surgery and receiving stromal injections of either human AD-MSC or activated human AD-MSC (n = 9 each), none of the grafts survived over the follow-up period of 8 weeks, with a mean time to rejection of 29.3±4.8 days (median 26) and 21.8±3.3 days (median 19), respectively.

The time to rejection showed no statistically significant differences among the treated groups. Surprisingly, the two-month survival rate was significantly lower in the treated groups than in the control group (p<0.05) (Fig. 2A).

**Fig 3:**
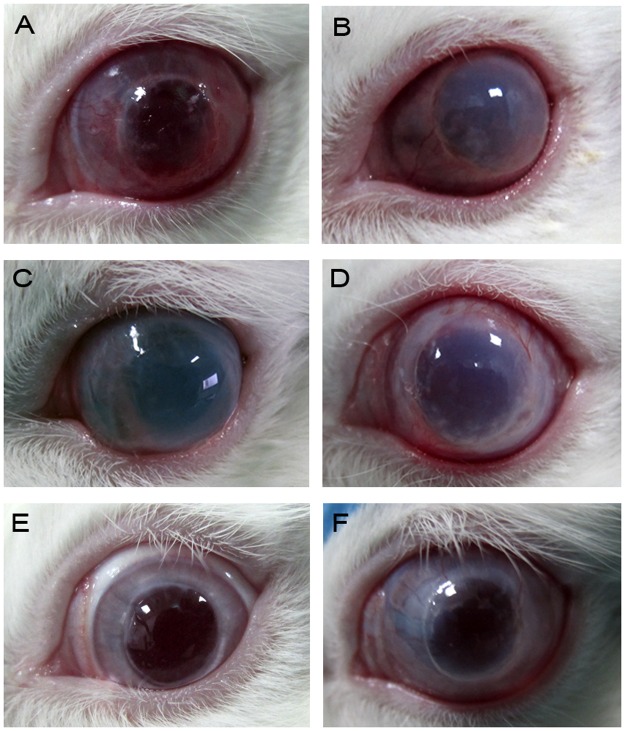
Representative photographs of transplanted corneas at the time of rejection. A) sham group; B) human AD-MSCs; C) activated human AD-MSCs; D) rabbit AD-MSCs after transplantation; E) unrejected cornea of one of the sham group rabbits; F) unrejected cornea of one of the MSC treated rabbits. Magnification 2x.

In order to discard possible immunological interactions of human AD-MSCs in the rabbits’ corneas, rabbit AD-MSCs were used in another set of experiments. However, in the rabbits subjected to transplant surgery that received stromal injections of rabbit AD-MSCs (n = 9), none of the grafts survived over the follow-up period of 8 weeks, with a mean time to rejection of 26.5±4.5 days (median 22). The 2-month survival rate was significantly lower than that of the sham group (p<0.05) and the time to rejection was similar to that of the human AD-MSC treated groups (p<0.05) (Figs. 2A and 3).

The clinical manifestation of rejection was characterized by vascularization of the graft followed by edema and corneal opacity (Fig. 3). Rejection index comparisons during the first days post-transplant demonstrated that transplantation affected all groups equally, suggesting that the transplant surgical technique was similar in all groups. The comparisons also showed that day 11 was predictive of rejection, that is, corneas that had an RI = 0 at day 11, would not be rejected later, even when sutures were to be removed at day 14. It also demonstrated that the event of suture removal at day 14 promoted a general worsening in all groups, but it did not affect the survival outcome. It is notable that injection of cells into the stroma was a statistically significant additional injury event in all the injected groups (Fig. 2B-2E).

**Fig 4:**
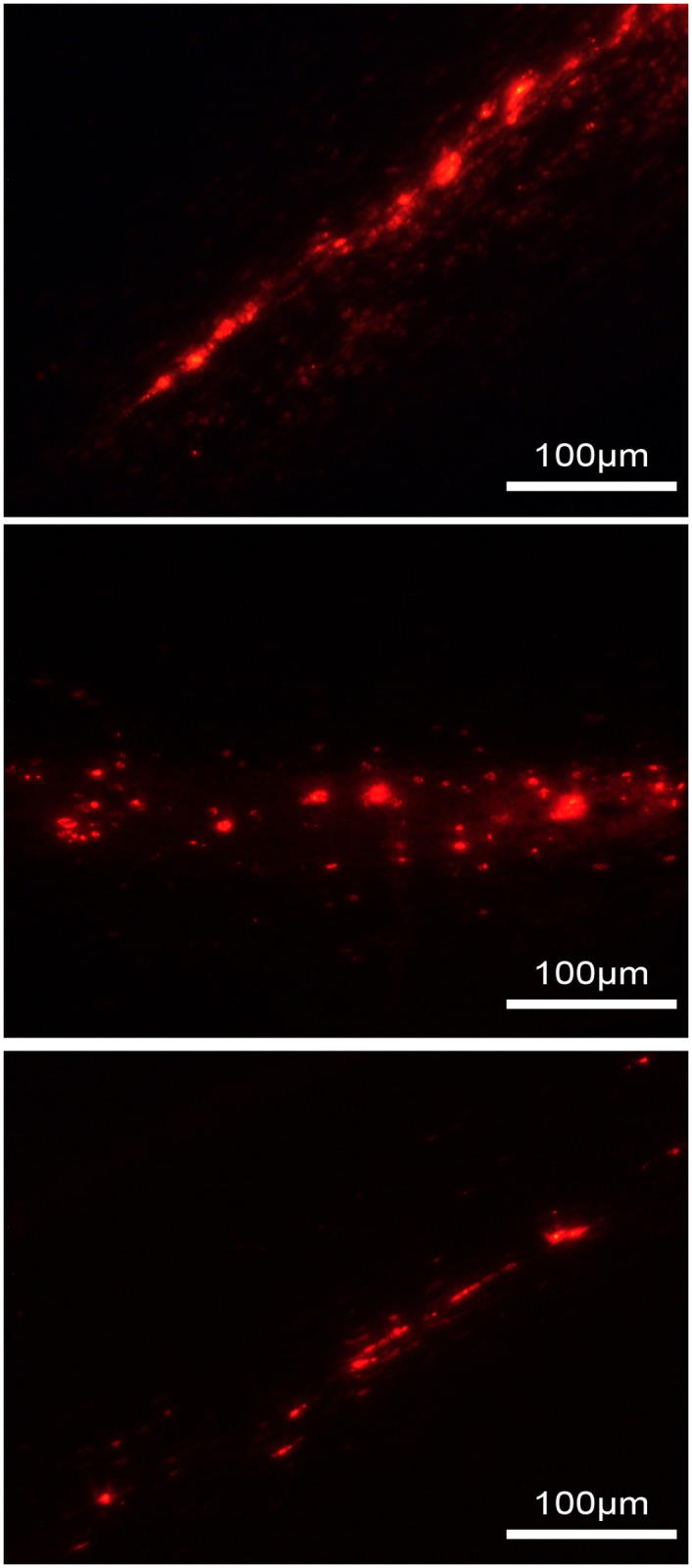
Fluorescently CM-DiI labeled MSCs remain in the corneal stroma even after graft rejection. A) human AD-MSCs; B) activated human AD-MSCs; and C) rabbit AD-MSCs are localized in different layers in the corneal stroma around the site of injection.

Human AD-MSCs activated or not, as well as rabbit AD-MSCs, were found in all the corneas treated with stromal injections of stem cells, but not in the sham injected ones. These MSCs were found in the host corneal stroma, located in multiple layers exclusively around the site of injection and present until the moment of euthanasia, which was up to 55 days in human nonactivated AD-MSCs, 47 days in human activated AD-MSCs, and 52 days in rabbit AD-MSCs (Fig. 4).

**Fig 5:**
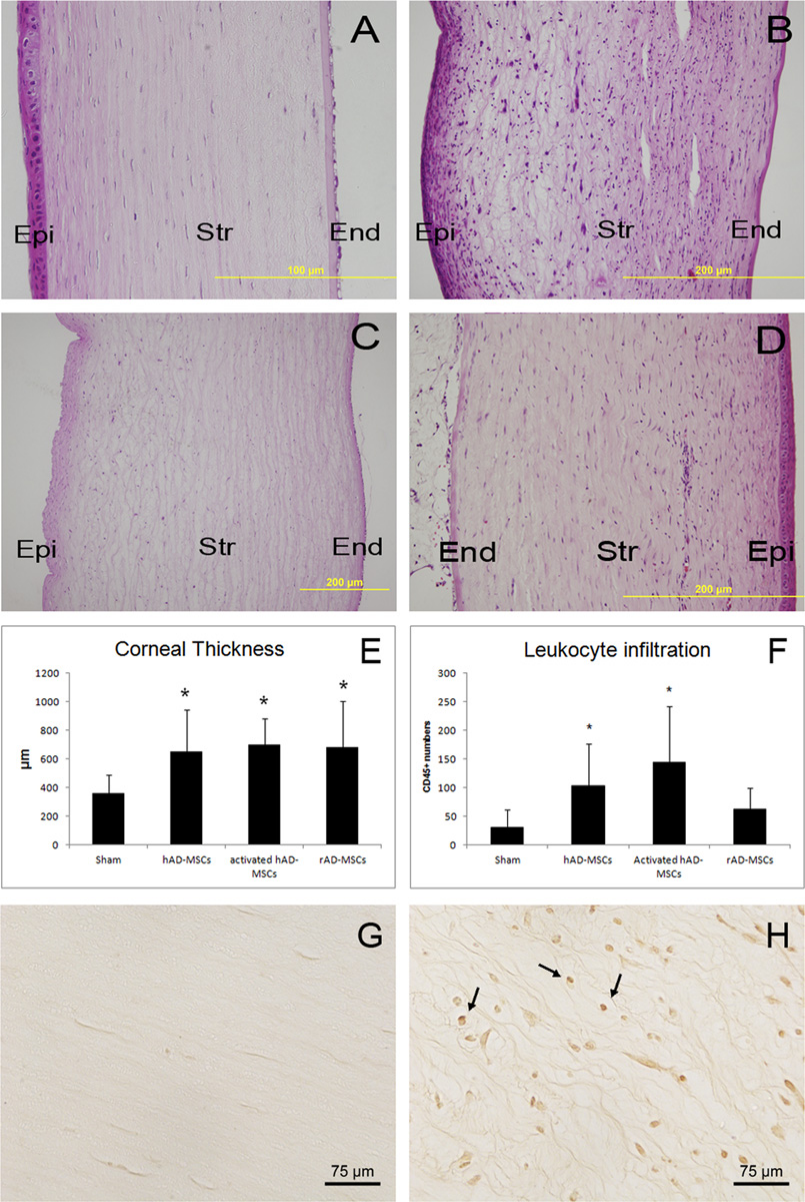
Representative photographs of histological sections of transplanted corneas. A) sham group; B) human AD-MSCs; C) activated human AD-MSCs; and D) rabbit AD-MSCs. Note the intense edema causing thickening of the corneal stroma (Str) in all MSC treated corneas, as well as leukocyte infiltration in the stroma. Epi: corneal epithelium; End: corneal endothelium. Hematoxylin and eosin staining. E) Corneal thickness measurements in the transplanted corneas. Stars indicate statistical significance at the p≤0.05 level. F) CD45 leukocyte infiltration measurements. Stars indicate statistical significance at the p≤0.05 level. G) CD45 immunohistochemistry in a transplanted cornea with sham treatment showing no positive cells. H) CD45 immunohistochemistry in a AD-MSC treated transplanted cornea. Positive cells are labeled in brown (arrows).

In the corneas injected with AD-MSCs, the grafts showed greater edema compared with the sham group (which showed no edema at all) (Fig. 5A-5D). Measurements demonstrated statistically significant differences in cornea thickness by all types of AD-MSC injection respect to sham (Fig. 5E). Grafts of the corneas subjected to stromal injection of human AD-MSCs, activated or not, showed the highest level of infiltration (lymphocytes and eosinophils) compared with the less infiltrated corneas treated with rabbit AD-MSCs and the sham group, which showed little infiltration (Fig. 5A-5D). CD45 immunostaining and quantification showed increased CD45+ cell numbers in corneas injected with human AD-MSCs but not with rabbit AD-MSCs (Fig. 5F-5H). Neovascularization was absent or very low in the recipient corneas of the sham group but was plentiful in the activated human AD-MSCs and the rabbit AD-MSC group. The non-activated human AD-MSCs showed a moderate level of neovascularization in the recipient cornea. No vessels reached the graft in any of the experimental groups (Fig. 5A-5D).

### AD-MSC immunophenotypical markers characterization

**Fig 6:**
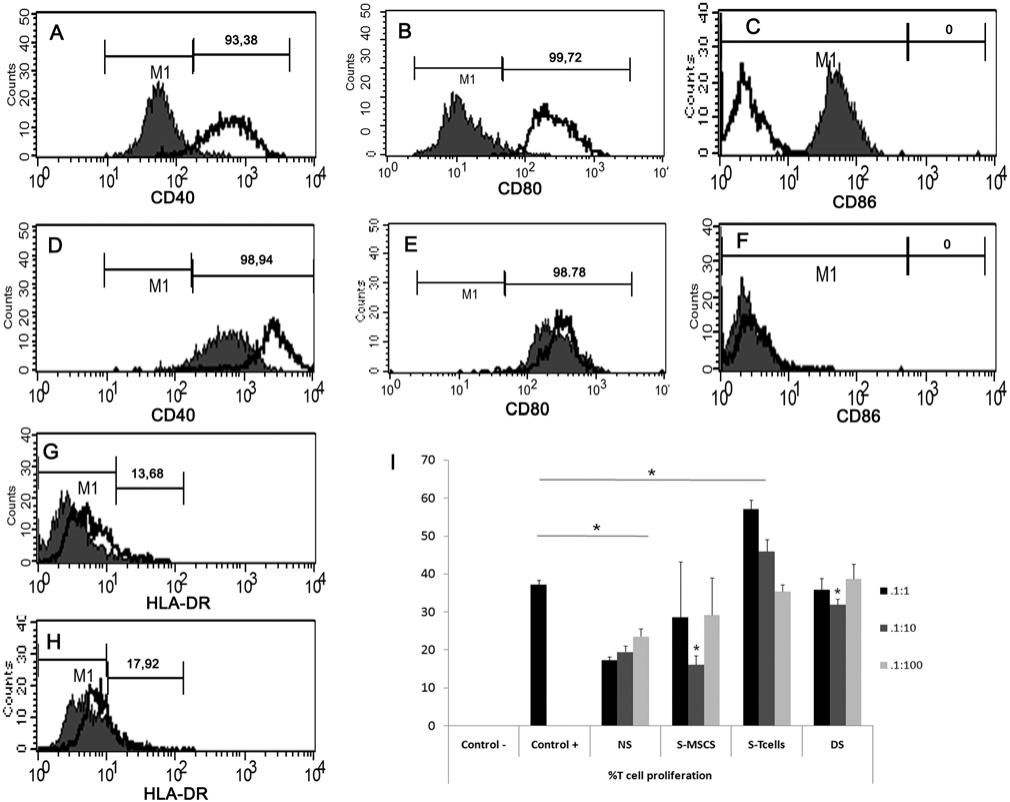
AD-MSC immunophenotypical markers and in vitro immunosuppressive ability characterization. A), B), C), G:) Expression of costimulatory molecules in naive AD-MSCs (Filled: control isotype; line: sample). D), E), F), H): Expression of costimulatory molecules after stimulation with INF-γ and TNF-α (Filled: naive; line: upon stimulation). I): AD-MSCs effect on T cell proliferation (expressed in %) at ratios MSC:T cells 1:1, 1:10 and 1:100. NS: non stimulated cocultures; S-MSCs: stimulated AD-MSCs, S-T cells: stimulated T cells; DS: double stimulation (T cells and AD-MSCs). Asterisks: statistically significant p≤0,05.

We immunocharacterized AD-MSCs for costimulatory molecules as in Menard et al. 2013 [22]. Our AD-MSCs constitutively expressed CD40 (Fig. 6A) and CD80 (Fig. 6B) but not CD86 (Fig. 6C). HLA-DR was expressed by a small percentage of AD-MSCs (Fig. 6G). Upon stimulation, the expression of costimulatory molecules increased greatly for CD40 and slightly for HLA-DR (Fig. 6D and 6H), but not for CD80 and CD86 (Fig. 6E and 6F).

### *In vitro* AD-MSCs immunomodulatory properties on T lymphocytes

All T cells of the negative control without any activation signal died within the follow-up period (Fig. 6I) as expected [23]. Proliferation of positive control was 37,1% (±1,3). AD-MSCs promoted T cells survival respect to negative control, but they were not able to reach the level of positive control (mean proliferation 17,2% ±1 in the 1:1 ratio). Increase in the T cell proliferation (mean 23,5% ±2) was achieved at higher ratios (1:100), being statistically significant respect to lower ratios. Stimulated AD-MSCs increased T cells proliferation up to positive control levels at ratios of 1:1 and 1:100 but not at 1:10. When stimulated T cells faced non stimulated-AD-MSCs their proliferation reached the highest levels (mean of 57,1 ± 2,4 at 1:1 ratio). At a ratio of 1:100, proliferation did not differ from that of positive control. The double stimulated group reached the same proliferation levels as those of positive control except for the 1:10 ratio.

### AD-MSC immunomodulatory secretion characterization

**Fig 7:**
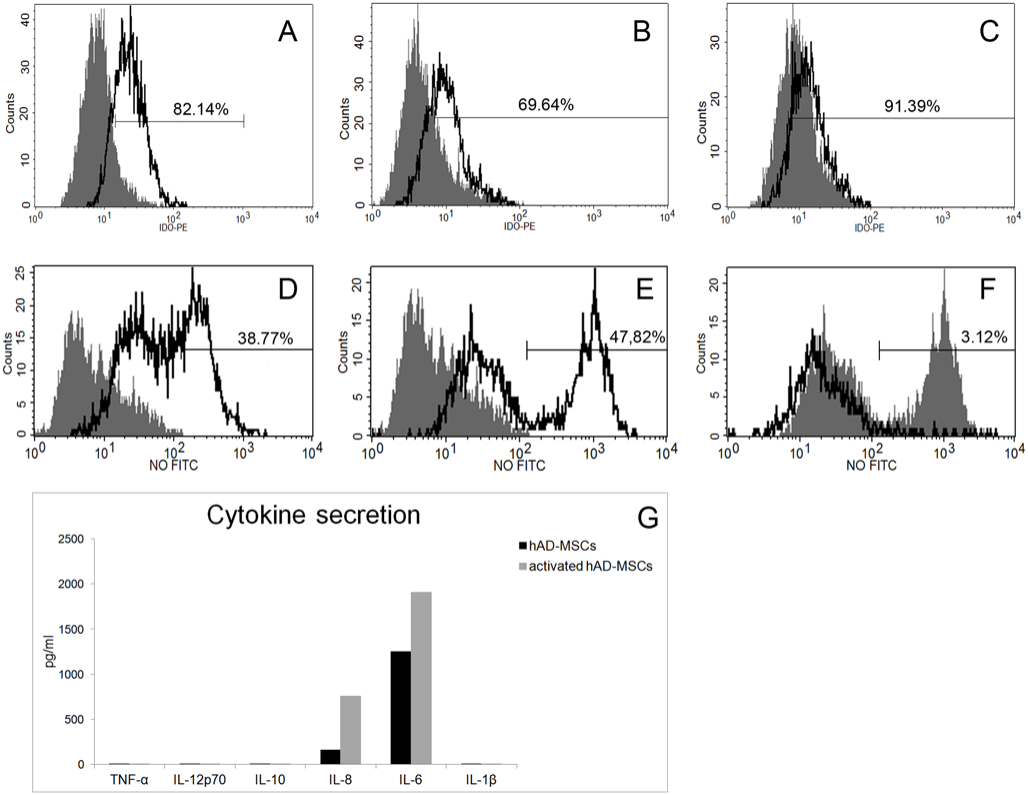
AD-MSC immunomodulatory secretion characterization. A) IDO production by rabbit AD-MSCs with respect to isotype control. B) IDO production by human AD-MSCs with respect to isotype control. C) IDO production by activated human AD-MSCs with respect to unactivated ones. D) NO production by rabbit AD-MSC respect to isotype control. E) NO production by human AD-MSCs with respect to isotype control. F) NO production by activated human AD-MSCs with respect to unactivated ones. G) Cytokine expression of human unactivated or activated AD-MSCs.

Immunophenotyping of rabbit, human and activated human MSCs showed that immunosuppressive molecule indoleamine (IDO) was produced by both rabbit and human AD-MSCs, but very slightly or not increased by TNFα and IFNγ activation (Fig. 7A-7C). With respect to immunosuppressive nitric oxide (NO), it was again detected in rabbit AD-MSCs (Fig. 7D) and in two subpopulations (about half of the cells with low and the other half with high expression) of human AD-MSCs (Fig. 7E). Unexpectedly, TNFα and IFNγ incubation did not stimulate NO production, but in contrast, all cells were found in the low-NO range (Fig. 7F). This result could partially explain why we did not obtain better results with activated cells respect to inactivated ones.

To further prove activation of AD-MSCs as well as immunomodulatory molecules secretion, we immunophenotyped our cells for TNFα, IL1β, IL-6, IL-8, IL-10 and IL-12. Among these, both normal and activated AD-MSCs produced only proinflammatory cytokines IL-6 and IL-8, with a moderate increase by TNFα and IFNγ incubation (Fig. 7G).

### High-risk grafts

We speculated that the high survival of normal-risk grafts (55%) could be masking the effect of the treatment with AD-MSCs in the experimental groups. We designed another cornea transplant model with a high risk of graft rejection by promoting vascularization by suturing the cornea previous to the transplant, which in humans is associated with higher rates of rejection.

**Fig 8:**
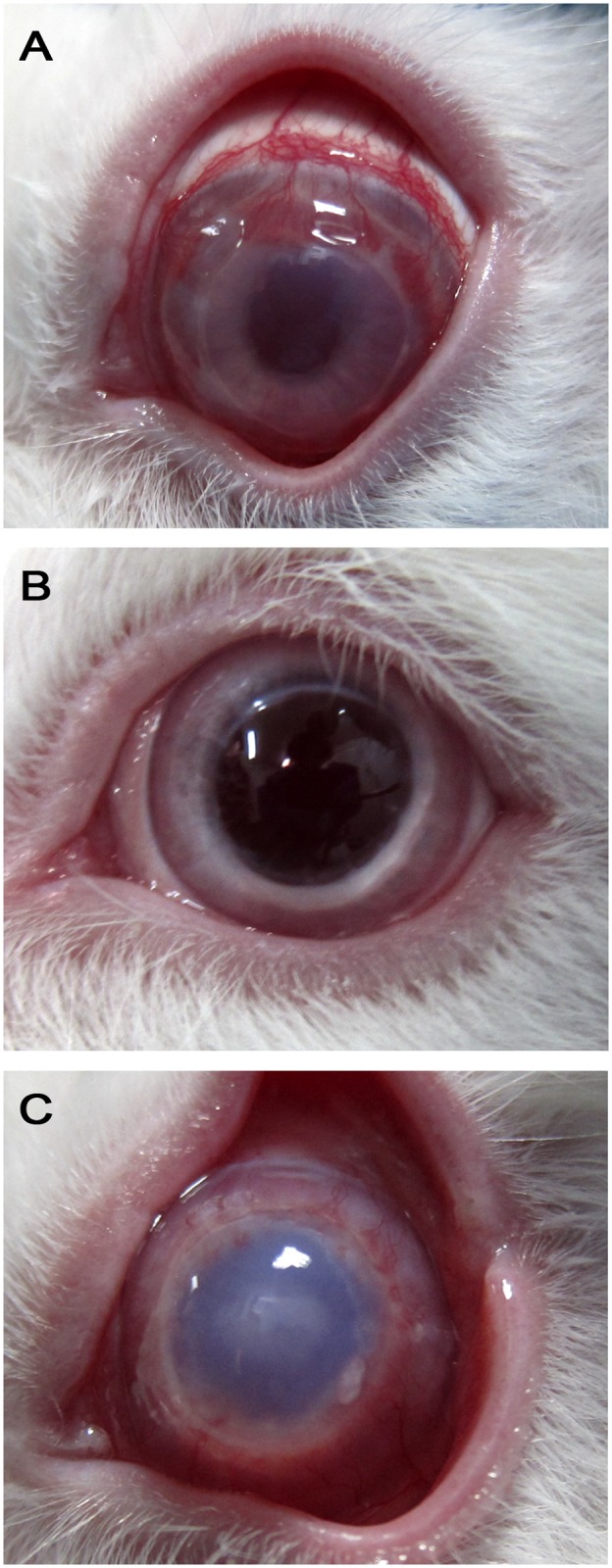
Representative photographs of the corneas of the high-risk model of corneal transplantation. A) prevascularization of the corneas, showing profusion of blood vessels invading the cornea; B) sham group of intravenous injection of vehicle, showing recovering of prevascularization and transparent cornea; and C) rabbit AD-MSC intravenous injection showing opacity of the cornea and rejection. Hematoxylin and eosin staining.

Within two weeks of placing the sutures, prevascularization was achieved in 3 to 4 of the cornea quadrants in all the animals (Fig. 8A).

As in the previous model the intrastromal injection of AD-MSCs was determined as an extra injury event, we injected the cells intravenously into this model. Also, as rabbit AD-MSCs demonstrated to promote less leukocyte infiltration but considerable secretion of immunosuppressive molecules IDO and NO, only rabbit AD-MSCs were employed.

**Fig 9:**
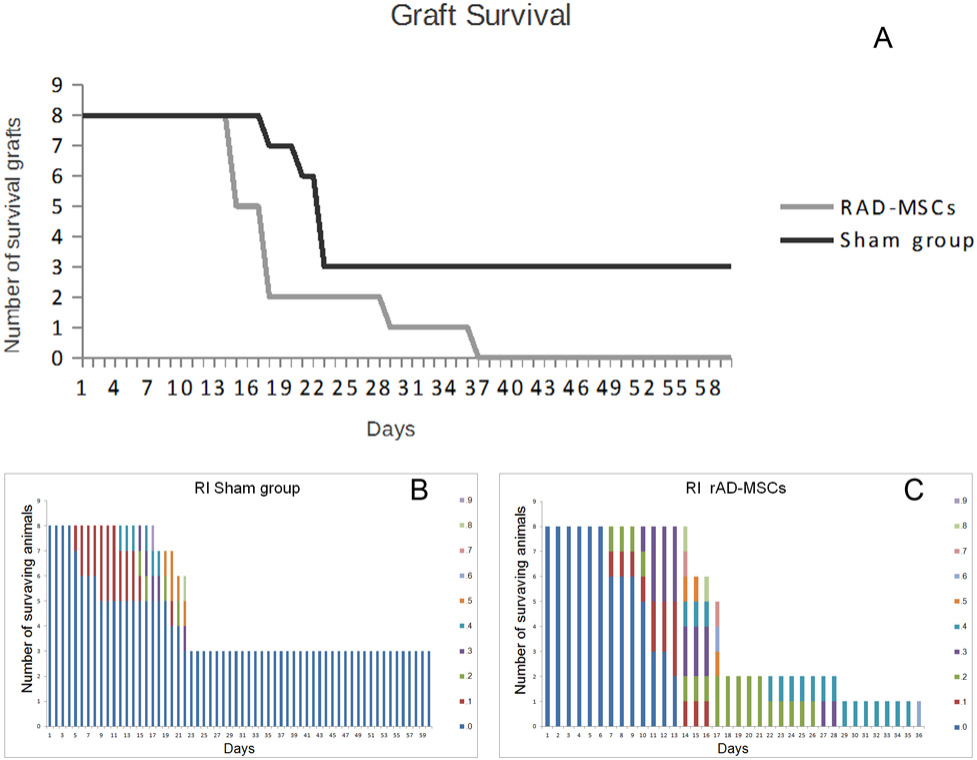
A) Survival curve of the corneal grafts of the high-risk model of corneal transplant. Graft survival was significantly shortened by intravenous injection of rabbit MSCs (rAD-MSCs) compared with vehicle injection (sham group), p = .085. B,C) Rejection index score (haze, edema, and neovascularization) comparison between groups. Different colors show different rejection index scores in the surviving grafts at any time point.

In the sham rabbits, just 3 of 8 grafts survived beyond the follow-up period, with a two-month survival rate of 37.5% (median = 22) (Fig. 9A). This demonstrated that prevascularization in our model was capable of reducing cornea transplant survival, even though the same strain of rabbits was used for donors and recipients (compare control groups in Fig. 2A and Fig. 9A). However, in the rabbits that underwent intravenous injection of rabbit AD-MSCs after cornea transplantation, none of the grafts that received AD-MSCs survived over the following period of 8 weeks (n = 8), with a mean time to rejection of 19.6±2.8 days (median 17) (Fig. 9A). The 2-month survival rate was significantly lower than that of the sham group (p<0.05).

The clinical manifestations of rejection were characterized by vascularization of the graft followed by edema and corneal opacity (Fig. 8). Rejection index comparisons demonstrated that transplantation affected both groups equally, suggesting that the transplant surgical technique was similar in both groups. Interestingly, intravenous injection of cells was not a statistically significant injury event (Fig. 9B, 9C). It also demonstrated that the event of suture removal at day 14 promoted a general worsening in both groups that affected the survival outcome, suggesting it is inadvisable to remove sutures in a similar future scenario.

**Fig 10:**
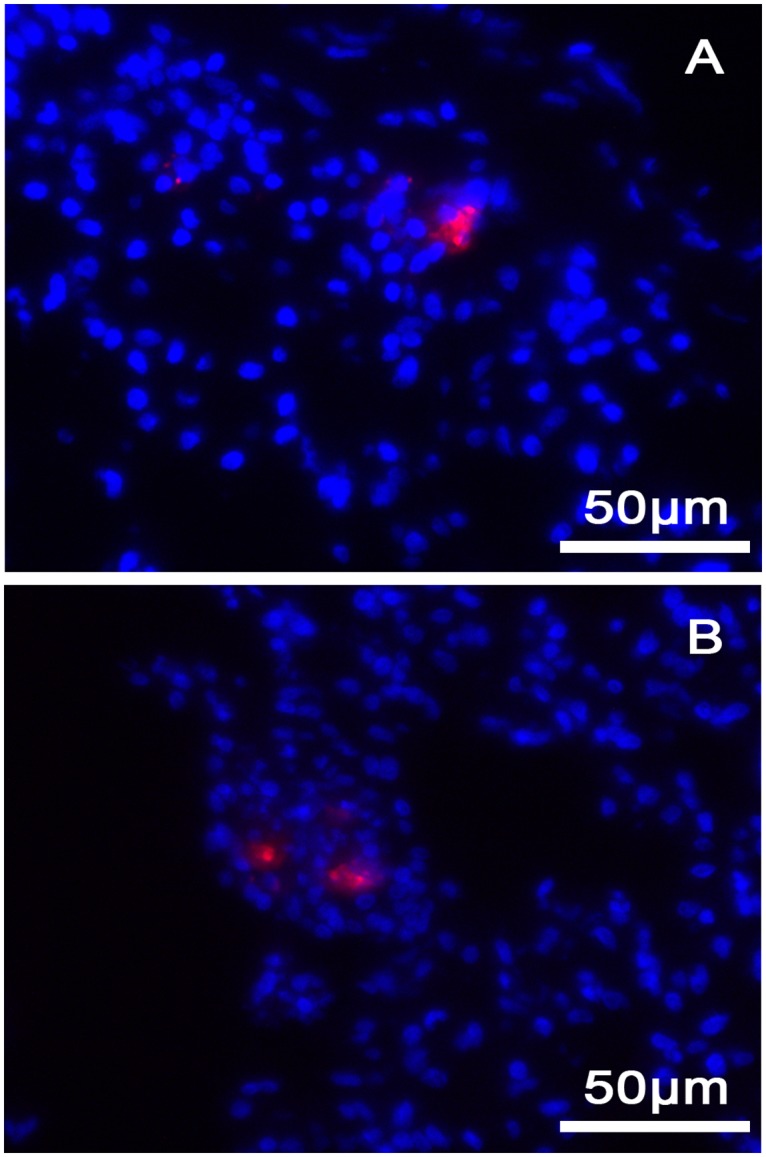
Fluorescent images of the lungs of two AD-MSC-injected rabbits showing the CM-DiI labeled cells in red. The DAPI nuclear counterstain is shown in blue.

AD-MSCs were not found in the corneal stroma in the rabbits injected intravenously, but were fluorescent in the lungs of the injected rabbits for at least 4 weeks after the injection (Fig. 10).

**Fig 11:**
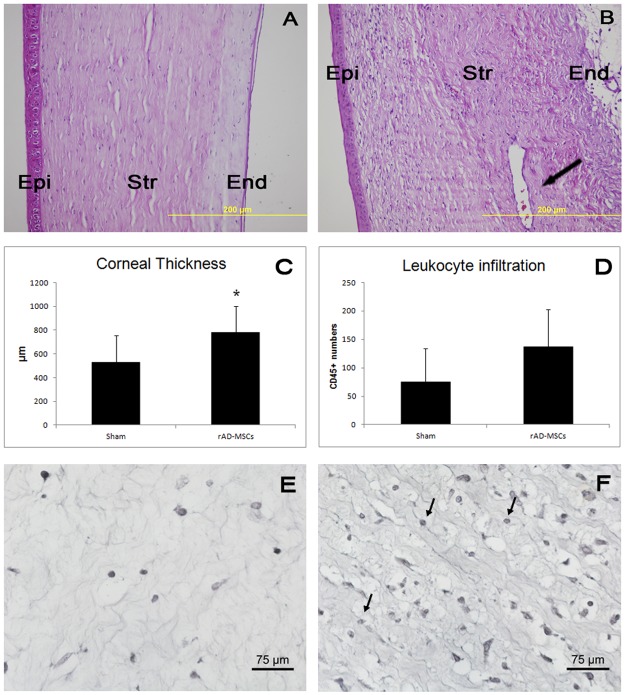
Representative photographs of histological sections of the transplanted corneas of the high-risk model of corneal transplant. A) sham group; B) rabbit AD-MSCs. Note the intense edema causing thickening of the corneal stroma (Str), and leukocyte infiltration in the stroma and blood vessels (arrows). Epi: corneal epithelium; end: corneal endothelium. Hematoxylin and eosin staining. C) Corneal thickness measurements in the transplanted corneas. Stars indicate statistical significance at the p≤0.05 level. D) CD45 leukocyte infiltration measurements. No statistical difference. E) CD45 immunohistochemistry in a transplanted cornea with sham treatment. F) CD45 immunohistochemistry in an AD-MSC-treated transplanted cornea. Positive cells are labeled in black (arrows).

The sham group showed less edema both in the graft and the recipient cornea, showing statistically significant differences in cornea thickness by AD-MSC injection (Fig. 11C). With respect to leukocyte infiltration, CD45 immunostaining and quantification showed slightly but not statistically significant increased CD45+ cell numbers in the injected versus sham corneas (Fig. 11D-11F). Neovessels appeared in the recipient corneas of the sham group and were increased in the rabbit AD-MSC group. These vessels did not reach the grafts in any case (Fig. 11).

**Fig 12:**
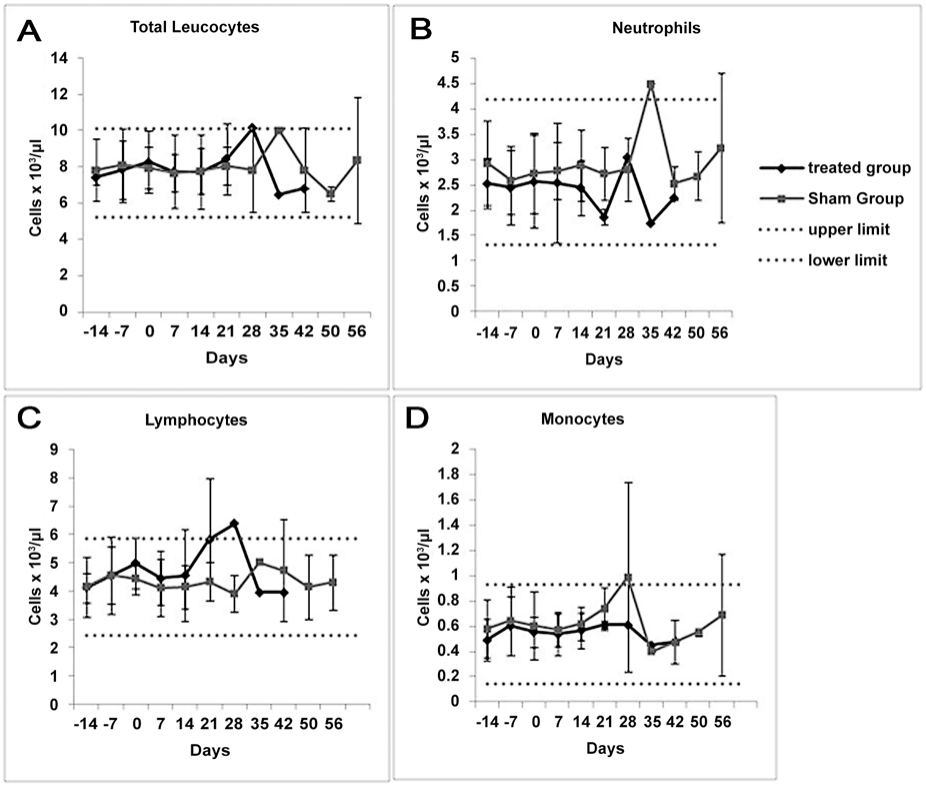
Differential blood parameters in the rabbits that underwent intravenous injection of rabbit AD-MSCs versus vehicle. The horizontal lines represent the normal range established as mean ± 2 SD of blood parameters in our rabbit population using data from day-14 of the experiment, when the rabbits were untouched. No statistical differences were found in any of the parameters analyzed between the sham and MSC-treated groups at any time point.

To better understand the mechanism of AD-MSC action on blood cell parameters in vivo, we examined differential leukocyte counts in the intravenously injected rabbits every week during the experimental period. As shown in the leukograms of Fig. 12, there were no significant differences between the treated and sham groups (p≤0.05) concerning leukocytes, neutrophils, monocytes, or lymphocytes.

## DISCUSSION

Over the last decade, attention has been drawn to the use of MSCs as a therapeutic tool in cell and solid organ transplantation to promote graft enhancement and decrease rejection. MSCs were first used successfully in a baboon model of skin allograft transplantation [24] and were further explored in other models of solid organ transplantation, such as the heart [25], the pancreatic islets [26], and the kidney [27]. Corneas are an ideal tissue in which to study graft behavior after MSC injection because the cornea allows direct access to examination, and due to its immune privilege, rejection times are delayed and systemic immunosuppression is not necessary, so the local effect of the grafted cells alone can be observed. In the present study, we investigated the effect of AD-MSC pre-treatment and/or as an adjuvant to the surgery on the modulation of rejection response in normal- and high-risk models of rabbit corneal transplantation.

In our normal-risk transplant model, we found that in contrast to our expectations, the injection of AD-MSCs into the graft junction at the time of surgery resulted in the induction of increased signs of inflammation, such as corneal edema and a higher level of infiltration of lymphocytes and eosinophils. This inflammation led to a lower survival of the graft compared with the sham-treated corneal transplants. It has been speculated that MSCs could be recognized by the adaptive immune system *in vitro* [28] and be immunogenic in an allogenic host and stimulate donor graft rejection *in vivo* in bone marrow transplants [29]. We speculated that the higher rejection rate in our model of normal risk and intrastromal AD-MSC rejection could be due to the use of human MSCs, creating a **xenogeneic environment**. However, we found no differences when using human or rabbit MSCs, suggesting that in the cornea this is probably not the primary cause. Other explanations could include the neovascularization/inflammation caused by the transplant, which breaks the barrier providing the corneal immune privilege, which is primarily due to the absence of blood vessels. In fact, in a previous study by our group in which the cornea was damaged by laser, thus preventing **inflammation**, we demonstrated that human AD-MSCs injected into the rabbit corneal stroma for regenerative purposes did not induce a rejection response [14]. However, in the current scenario in which an inflammatory environment is already established, the secretion of IL-6 and IL-8 by AD-MSC can exacerbate the chemotaxis of leukocytes to the injected zone, thus increasing inflammation. In this case the action of pro-inflammatory cytokines IL-6 and IL-8 seems to be higher than the immunosuppressive effects of IDO and NO also secreted by AD-MSCs.

In addition, it is feasible that other paracrine secretions of AD-MSCs, such as the secretion of angiogenic cytokines including VEGF, HGF, and FGF2, which are known to induce **neovascularization** [30,31], abrogate the corneal immune privilege. Again, the secretion of the pro-angiogenic cytokine IL-8 by our AD-MSC correlates with our finding of increased neovascularization with respect to the sham injection. Induction of neovascularization is a well-known effect of MSCs, as proven in myocardial infarction treatment [31]. This effect in such a setting, which might be useful for promoting blood supply and treating myocardial infarction, is a well-known high risk factor for corneal rejection. In our study, neovascularization was associated with rejection in some cases. We hypothesize that a local inflammatory environment is not suitable for the MSCs to exert their immunosuppressive effect on the early inflammatory response [9].

**Activation of AD-MSCs** with IFN-γ and TNF-α caused an even higher rate of rejection. MSCs depend on the presence of these cytokines to exert their immunomodulatory effect [19,20,32], theoretically promoting graft survival; however, we found the opposite. MSCs need accurate cytokine regulation, and IFN-γ and TNF-α can turn MSCs from an anti-inflammatory to a pro-inflammatory pattern and vice versa [33–37]. The MSCs primed with IFN-γ have been shown to upregulate MHC-I, MHC-II and CD40 [20,36,38], becoming non-professional antigen presenting cells, which could activate local T-cells thus increasing the rejection rate. In fact, when we immunocharacterized AD-MSCs for costimulatory molecules as in Menard et al. [22], we found that our AD-MSCs constitutively expressed CD40 and CD80, and also HLA-DR in a small percentage of AD-MSCs. Upon stimulation, the expression of CD40 increased greatly and slightly for HLA-DR. In Menard et al. [22], MSC were negative for both CD40 and CD80, but both markers increased upon stimulation. Importantly, these differences in unstimulated cells could be due to differences in donor sample, as it has been recently demonstrated that MSC isolated from different donors varied widely in their efficacy in modulating inflammation in a mouse model of chemical injury to the cornea [39].

In our in vitro studies of AD-MSC-T cell interaction, we demonstrated that AD-MSC promoted T cell survival and proliferation even when unstimulated, so in contrast to previous studies, they demonstrated no immunosuppressive ability. When stimulated T cells faced non stimulated-AD-MSCs their proliferation reached the highest levels suggesting that stimulated T cells exert a feedback loop stimulating the proinflammatory phenotype of AD-MSC.

Respect to secreted immunosuppressive molecules, in our case, IDO and NO were detected in both rabbit and human AD-MSCs, but were only slightly or not increased by TNF-α and IFN-γ. This could partially explain why we did not obtain better results with activated cells respect to inactivated ones. In our case, the increased secretion of pro-inflammatory cytokines IL-6 and IL-8 in activated AD-MSC respect to unactivated ones could partially explain the poorer results obtained with activated AD-MSC.

In other studies [40], MSCs derived from bone marrow that were systemically administered post-transplant between two rat strains improved corneal graft survival. Apart from the species differences, in this case the MSCs were from the donor, whereas in our case they were from the recipient, as we aimed to use AD-MSCs in an autologous context for patient use in the long term. The source of the MSCs has not been clearly described in terms of its immunosuppressive properties. MSCs derived from adipose tissue have been shown to exhibit immunosuppressive capabilities both *in vitro* [41] and *in vivo* [42,43] and have been clinically used for treatment of graft versus host disease in humans [44]. Nevertheless, the detrimental effects of MSCs derived from bone marrow have been described in a kidney transplant model [45] and a heart transplant model [10]. Jia et al. [40] achieved the best results when co-administering cyclosporine A at 2 mg/kg, but surprisingly, when the dose was lowered to 1 mg/kg, they found a negative effect. This could be due to the immunosuppressive environment created by the high dose of cyclosporine, which in turn leads MSCs to exert anti-inflammatory functions and vice versa. An immunosuppressed setting is known to help MSCs exert their immunomodulatory activity [46,47], as shown in graft versus host disease (GvHD) trials in which patients are not immunocompetent [44,48–50]. Similar to our findings, some authors reported accelerated rejection of graft cells in mice when previously injected with allogenic MSCs in a model of bone marrow transplant [51].

In another model of corneal rejection using two strains of mice and systemic injection of human MSCs [9], the MSCs prevented rejection by aborting the early inflammatory response. Discrepancies between these studies and ours might be caused by differences in the experimental parameters, including the animal model, the time of injection, the immunosuppression, and the mode of delivery, among others.

All the above studies have been performed in normal- or low-risk transplant settings. As stated in the introduction, the primary problem to be solved in corneal transplantation involves high-risk recipients. In our high-risk transplant model, in which immune ocular privilege was undermined by the induction of neovascularization prior to graft surgery, we found that the use of systemic rabbit AD-MSCs prior, during, and at different time points after the surgery resulted in a lower survival rate of the graft compared with non-treated corneal grafts. Similarly, other authors [52], in a pig to rat model more similar to a high-risk human transplant, found that rat allogeneic MSCs administered topically did not prolong the survival of the corneal graft.

In our high-risk study, intravenously administered MSCs were not found in the cornea, so these cells must systemically produce some factors that accelerate graft rejection. Similar to our results, some authors have reported a near absence of MSCs in the cornea after being systemically injected [9]. Again, angiogenic factors such as VEGF and IL-8 secreted systemically by the AD-MSCs resident in the lungs could be the culprit in terms of neovascularization and higher rejection rates.

In addition, it is known that MSCs are able to induce tolerogenic dendritic cells (DCs) [53–55] and to increase regulatory T lymphocytes [25,46,56]. We administered syngeneic rabbit MSCs intravenously 7 days before surgery to induce a systemic tolerogenic environment, and on days 0, 3, and 14 to maintain it. Other studies in low-risk settings [9,40] found positive results when the cells were administered after surgery, or when the cells were injected 1 day before and the day of the surgery. In these cases, the mechanism of action could not be tolerogenic, because they achieved their positive results with post surgical or peri surgical administration.

The models we present in this study are more similar to human corneal transplants. The similar size of the eyes allows for standard corneal transplant surgery, including the number of sutures, the type of sutures, the type of trepanation, and the size of trepanation, whereas using other, smaller animal models imposes the need to adapt the surgical technique. In addition, corneal allografts in rats are normally rejected within two weeks of transplant [57], whereas in mice [9] syngeneic transplant allows 100% survival of the graft at 42 days, and allogenic transplant promotes 60% rejection earlier than a month post-transplant. In our experiments with rabbits, more than 50% of the corneal allograft often survived, even without immunosuppression, for over 2 months, closer to what is seen in humans if no immunosuppression is given. However, in both of our rabbit models, there were considerable differences in the clinical signs of rejection from our experience in humans. The surgery is more difficult due to the higher elasticity of the rabbit cornea, the higher retraction after trepanation, and a smaller anterior camera that provokes more frequent anterior synechiae and post-transplant ocular hypertension. Thus although these animal models can reveal many insights into the mechanism of corneal graft rejection and the effect of different treatments, the extrapolated results must be handled with care in their translation to clinical practice.

Based on our results, local or systemic treatment with AD-MSCs to prevent corneal rejection in rabbit corneal models of normal or high risk of rejection does not increase survival but rather can increase inflammation and neovascularization, and undermine the innate ocular immune privilege. Parameters including the risk of rejection, the inflammatory/vascularization environment, the source of the cells, the time of injection, the immunosuppression, the number of cells, and the mode of delivery must be determined before translating the possible benefits of the use of MSCs in corneal transplant to clinical practice.
